# A Drug Discovery Pipeline for MAPK/ERK Pathway Inhibitors in *Caenorhabditis elegans*

**DOI:** 10.1158/2767-9764.CRC-24-0221

**Published:** 2024-09-18

**Authors:** Szymon Gorgoń, Ola Billing, Anna U. Eriksson, Oskar Hemmingsson

**Affiliations:** 1 Department of Diagnostics and Intervention, Surgery, Umeå University, Umeå, Sweden.; 2 Chemical Biology Consortium Sweden, Umeå University, Umeå, Sweden.; 3 Wallenberg Centre for Molecular Medicine, Umeå University, Umeå, Sweden.

## Abstract

Oncogenic signaling through the MAPK/ERK pathway drives tumor progression in many cancers. Although targeted MAPK/ERK pathway inhibitors improve survival in selected patients, most tumors are resistant. New drugs could be identified in small-animal models that, unlike *in vitro* models, can address oral uptake, compound bioavailability, and toxicity. This requires pharmacologic conformity between human and model MAPK/ERK pathways and available phenotypic assays. In this study, we test if the conserved MAPK/ERK pathway in *Caenorhabditis elegans* could serve as a model for pharmacological inhibition and develop *in vivo* pipelines for high-throughput compound screens. Using fluorescence-based image analysis of vulva development as a readout for MAPK/ERK activity, we obtained excellent assay *Z*-scores for the MEK inhibitors trametinib (*Z* = 0.95), mirdametinib (*Z* = 0.93), and AZD8330 (*Z* = 0.87), as well as the ERK inhibitor temuterkib (*Z* = 0.86). The throughput was 800 wells per hour, with an average seed density of 25.5 animals per well. Readouts included drug efficacy, toxicity, and pathway specificity, which was tested against pathway activating upstream (*lin-15*)- and downstream (*lin-1*) mutants. To validate the model in a high-throughput setting, we screened a blinded library of 433 anticancer compounds and identified four MEK inhibitors among seven positive hits. Our results highlight a high degree of pharmacological conformity between *C. elegans* and human MAPK/ERK pathways, and the presented high-throughput pipeline may discover and characterize novel inhibitors *in vivo*.

**Significance:**

Many tumors depend on MAPK/ERK signaling to sustain growth, avoid cell death, and metastasize. We show that specific and clinically relevant MAPK/ERK signaling inhibitors can be discovered *in vivo* with a high-throughput screening pipeline in small animals.

## Introduction

Evolutionary conserved signaling pathways involved in proliferation, cell growth, or apoptosis are often altered in cancers (1). One of these is the RAS-RAF-MEK-MAPK/ERK (MAPK/ERK) pathway, which is hyperactivated in 85% of all human cancer tumors (2). Over the last two decades, inhibitors have been developed against actionable wild-type (WT) and mutant pathway proteins. Targeted drugs are available against several receptor tyrosine kinases and RAF and MEK proteins, and the first inhibitor against KRAS^G12C^ was recently approved (3). These have, together with advances in immunotherapy, moved oncology toward personalized treatment with markedly improved patient outcomes in many cancers. One example is melanoma, in which combination therapy with BRAF and MEK inhibitors provides 34% overall 5-year survival in BRAF-activated metastatic melanoma (4). However, three major drawbacks remain for all current MAPK/ERK pathway inhibitors and highlight the need for better drugs. First, treatment with MAPK/ERK pathway inhibitors frequently causes severe adverse side effects, including secondary tumor development due to paradoxical activation of the targeted pathway (5). Second, early acquired or inherent resistance to treatment results in modest effects on overall survival (6–8). Third, most MAPK/ERK-activated cancers are still unresponsive to available inhibitors (6).

Screens for novel inhibitors are typically performed *in vitro* and then validated in vertebrate models (9)*. In vitro* assays are fast and cost-efficient but may generate numerous false positives. This is because they lack the physiological context of pharmacokinetics, metabolism, and tissue cross-talk (10, 11). *In vivo* assays, on the other hand, have the potential to validate oral uptake, membrane permeability, effectiveness, and toxicity at an early stage (12, 13). On the downside, screening assays performed in vertebrate models are tedious, expensive, and associated with ethical issues (13). Small-animal and invertebrate models have the potential to overcome some of the inherent drawbacks of both *in vitro* and vertebrate models (11–14) and seem useful when targeting evolutionary conserved pathways relevant in cancer, such as MAPK/ERK, Wnt, Notch, and insulin pathways (15–22).

**Figure 1:**
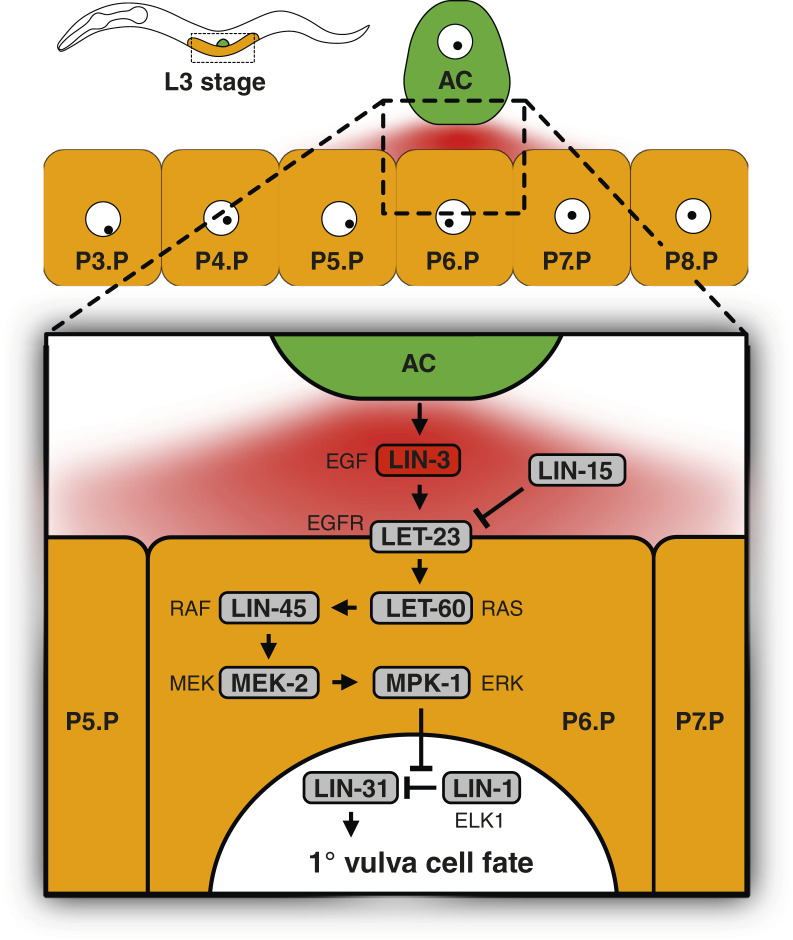
MAPK/ERK signaling promotes primary vulva cell fates. At the third larval stage (L3), the anchor cell secretes a LIN-3/EGF ligand gradient over six vulva precursor cells, each competent to adopt vulva cell fates. The central cell (normally P6.P) that receives the highest dose of LIN-3 will adopt the primary vulva cell fate. In the receiving cell, activated MPK-1/ERK phosphorylates the ETS domain–containing transcription factor LIN-1/ELK1, thereby disinhibiting the FOXB-type transcription factor LIN-31 that will promote the primary vulva cell fate. The two adjacent cells (normally P5.P and P7.P) will adopt secondary vulva cell fates due to lateral inhibition through WNT signaling from the primary vulva cell. The lateral-most cells (P3.P, P4.P, and P8.P) will avoid vulva cell fates and fuse with the hypodermis. Homologous human proteins are indicated next to *C. elegans* proteins. AC, anchor cell.

Proteins involved in MAPK/ERK signaling are conserved between the nematode *Caenorhabditis elegans* and humans (23). The *C. elegans* LIN-45 protein is 53% identical to human BRAF, and *C. elegans* MEK-2 and MPK-1 proteins are 52% and 81% identical to human MEK1 and ERK, respectively (24). In the nematode, MAPK/ERK signaling promotes vulva cell fates (Fig. 1). Aberrations to the MAPK/ERK signaling pathway are therefore readily scored *in vivo*: Inhibition results in a vulvaless (Vul) phenotype, and overactivity results in ectopic lateral cell induction and a multivulva (Muv) phenotype. If MAPK/ERK pathway gain-of-function mutants are exposed to a targeted inhibitor, their Muv phenotype may be rescued. This has previously been tested in strains expressing overactivated LET-23/EGFR (25), LIN-45^S312A/S453A^/BRAF (26), and LET-60^G13E^/RAS^G13E^ (27), implying that *C. elegans* could be used in large-scale drug screens for MAPK/ERK-inhibiting compounds. However, no assay suitable for large-scale screens has been reported.

The aim of this study was to develop and validate a high-throughput *in vivo* pipeline that can detect clinically relevant MAPK/ERK-inhibiting compounds in *C. elegans*.

## Materials and Methods

### Strains

The ST65 strain (RRID: WB-STRAIN:WBStrain00034489), harboring the integrated transgene *ncIs13[ajm-1::GFP]* and the N2 WT strain (RRID: WB-STRAIN:WBStrain00000001) was obtained from the *Caenorhabditis* Genetics Center, which is funded by NIH Office of Research Infrastructure Programs (P40 OD010440). Strains MT1001 [*lin-1(e1777)*; RRID: WB-STRAIN:WBStrain00026770] and VB985 [*lin-15(n765)*; WBVar00089736] were kindly provided by Simon Tuck. Nematode cultures were maintained at 20°C on agar plates seeded with *Escherichia coli* OP50 (28).

### Chemical compounds

We used the following compounds for the assay development: dabrafenib, encorafenib, temuterkib, and TAK-632 from MedChemExpress and vemurafenib, trametinib, mirdametinib, AZD8330, CC-90003, SCH772984, LB42708, sotorasib, lapatinib, erlotinib, mubritinib, 4EGI-1, 4E1RCat, and CP-724714 from Selleckchem. DMSO stocks were aliquoted and stored at −80°C until use. 2× working solutions were prepared prior to each experiment in S-complete medium [200 mL H_2_O, 1.17 g NaCl, 0.2 g K_2_HPO_4_, 1.2 g KH_2_PO_4_, 0.2 mL cholesterol, 2 mL 1 mol/L potasium citrate pH 6.0, 2 mL trace metal solution (1.86 g disodium EDTA, 0.69 g FeSO_4_ • 7 H_2_O, 0.2 g MnCl_2_ • 4 H_2_O, 0.29 g ZnSO_4_ • 7 H_2_O, and 0.025 g CuSO_4_ • 5 H_2_O dissolved in 1L of H_2_O), 0.6 mL 1 mol/L CaCl_2_, and 0.6 mL 1 mol/L MgSO_4_]. The final DMSO concentration was 0.5% in all conditions.

### Bacteria

Overnight 2X yeast extract tryptone (YT) cultures of *E. coli* strains OP50 (RRID: WB-STRAIN:WBStrain00041969) or streptomycin-resistant OP50-1 (RRID: WB-STRAIN:WBStrain00041971), grown in 50 μg/mL streptomycin, were aliquoted into 50-mL Falcon tubes, pelleted, and washed twice in S-complete medium. The pellet was resuspended in 18 mL S-complete medium, followed by 24-hour incubation at 20°C. The next day, S-complete cultures were diluted to ∼5.6 × 10^9^ cells/mL, poured out onto culture plates (max 6.34-cm water column), and killed by UV irradiation (3 × 15 minutes with manual shaking in between, 60 mJ/cm^2^ in total) using a CL-1000 UV crosslinker (RRID: SCR_019820). Cultures were then transferred to 4°C, and 100 μL was withdrawn and tested overnight at 37°C for viability. Cultures producing 10 bacterial colonies or less were considered sufficiently inactivated and were used as a 2× bacterial working solution.

### Worm synchronization

Mixed-stage worm cultures were rinsed off from three Ø 9-cm agar plates in M9 buffer, and adults were collected in a 40-μm mesh filter. These were washed twice in M9 and then bleached in freshly prepared bleaching solution (3.21 mL MQ water, 500 μL 5 mol/L NaOH, and 290 μL 16% sodium hypochlorite) for 10 minutes to release embryos. Embryos were pelleted (17,000 × *g*, 20 seconds) and washed twice in M9. Embryos were then hatched on agar plates without food and synchronized in the L1 stage for a maximum of 20 hours at 20°C. Next, L1 larvae were rinsed off in M9, sieved through a 40-μm mesh filter, and pelleted (17,000 × *g*, 1 minute). These were seeded onto three 9-cm plates and grown until adulthood with food for 3 to 4 days at 20°C.

#### Assays in mutant backgrounds

The now adult worms were rinsed and bleached as before to synchronize L1s. Synchronized L1s were collected in M9, filtered, and incubated for 1 hour at 20°C in 1 mL antibiotic solution (M9 buffer with 50 U/mL penicillin and 50 μg/mL streptomycin), followed by washing twice in S-complete medium. After the last wash, the L1 worm pellet was resuspended in 2× bacterial working solution (see the section on “Bacteria”) and diluted to approximately 25 worms per well. The now 2× bacteria/worm solution was mixed 1:1 with 2× chemical compound solutions (see the section on “Chemical compounds”).

#### Assay in WT background

Adults were rinsed and allowed to lay eggs in 10 mL staging medium (M9 buffer with 50 μg/mL streptomycin, 50 U/mL penicillin, 200 μg/mL carbenicillin, 10 μg/mL nystatin, and 10 μg/mL cholesterol) in a T25 cell culture flask at 20°C with gentle shaking for 20 hours. Hatched L1s were sieved as before and washed two times in M9 and, right before drug exposure, one more time in S-complete before they were diluted to around one animal/μL in 2× bacterial working solution.

### Detection assay

The ST65 strain was used. We dispensed 50 μL of worm/compound mix into each well in 96-well plates and incubated the animals for 4 to 5 days at 20°C until the DMSO control animals reached early adulthood. The animals were then sedated for 30 minutes by adding 4 μL 20 mmol/L levamisole per well before scoring. In each experiment, all compounds were run in 6-well replicates for each concentration. Each experiment was repeated three times.

#### Manual imaging and scoring

Green fluorescence images were captured using a Trophos Plate Runner HD machine (Trophos). Adults and larvae were discriminated based on pharyngeal size (GFP) and body size (autofluorescence). Large, intense GFP foci in the ventral mid-body were scored as vulvae.

#### Automated imaging

Using Molecular Devices ImageXpress High-Content Confocal Imaging System (RRID: SCR_020294), we acquired four image fields per well with 3 ms exposure time for brightfield using 60% transmitted light (TL60) and 30 ms for GFP. Field x- and y-distances were adjusted manually to avoid overlap. The laser autofocus was set to focus on the plate and well bottom at the first field only, with a post-laser z-offset of 87.48 μm for GFP and an additional z-offset of 15 μm for TL60, rendering typical acquisition times of 7 minutes per plate.

#### Image analysis

The analysis was performed using MetaXpress software (RRID: SCR_016654) and CellProfiler image analysis software (RRID: SCR_007358; see Supplementary Methods for details). Outlier wells averaging >1.5 vulvae/adult were inspected manually and removed if containing confounding artifacts, such as GFP signals from larvae or precipitates.

### Validation assays in mutant backgrounds

MT1001 [*lin-1(e1777)*] and VB985 [*lin-15(n765)*] strains were used. Experiments were set up similar to the ST65 protocol for detection of MAPK/ERK inhibitors, but with 160 μL of worm/compound mix per well. After the 4 to 5 days of incubation, all 12 replicate wells were pooled in a microcentrifuge tube and pelleted (6,500 × *g*, 1 minute). Worms in each pellet were sedated with 5 μL 20 mmol/L levamisole for 30 minutes. The worms were mounted on agarose pads and examined by Nomarski microscopy using Olympus BX51 Fluorescence Microscope (RRID: SCR_018949). Animals possessing big oocytes, embryos, a fully developed vulva, or cuticle alae were considered adults. Protruding structures in the ventral mid-body were scored as vulvae and pseudovulvae.

### Validation screen

For the validation screen, we bought a chemical library (SciLifeLab) with 433 oncology-related compounds (see Supplementary Table S1 for the full list), in which compounds had been acoustically dispensed from 10 mmol/L stocks into 96-well plates. DMSO concentrations were adjusted to 0.5% in all wells, including negative controls. Trametinib was used as a positive control at 7 μmol/L. All chemicals were kept at −20°C until use. All other experimental conditions, including automated imaging and MetaXpress-based image analysis were carried out as described previously (see image analysis). Primary hit selection: (i) Wells were excluded if they contained progeny or fluorescent speckles from drugs or bacteria, indicated by too many objects classified as vulvae. The cutoff was vulvae >2× the number of adults in the positive control. (ii) Wells were excluded they contained <30% adults compared with the positive control, indicative of severely reduced larval growth. (iii) Remaining compounds with vulvae/adult scores <0.4 were considered primary hits. Scoring of the Muv phenotype in adult *lin-1(e1777)* mutants was done manually from ImageXpress images.

### Statistics

For each genotype and drug treatment, mean values were calculated from at least three independent experiments during assay development. Assay quality was estimated by calculating Z-factors, as described previously. The screen was run with one datapoint per compound. To validate 1° hits, we ran two independent experiments with three concentrations per compound. Downstream testing for MAPK/ERK pathway specificity in the *lin-1(e1777)* background was performed in one single experiment. Heatmaps, simple regression analyses, and violin plots were calculated and generated using GraphPad Prism (RRID: SCR_002798) software.

### Data availability

The data generated in this study are available upon request from the corresponding author.

## Results

### Detection of MAPK/ERK signaling inhibitors *in vivo*

**Figure 2:**
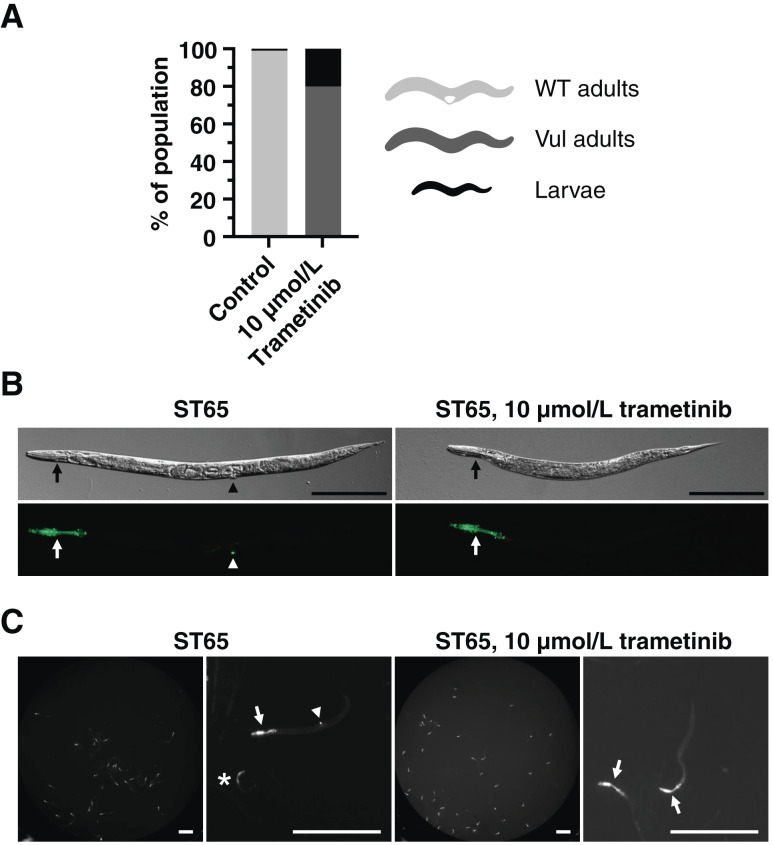
Scoring of vulva tissue. **A,** Bar graph showing distributions of indicated phenotypes in WT N2 animals after exposure to control DMSO media (*n* = 244) or 10 μmol/L trametinib (*n* = 296). **B,** Nomarski and fluorescence images of the ST65 strain, expressing AJM-1::GFP in pharyngeal muscle cells (arrows) and vulva tissue (arrowhead). The pharyngeal signal is retained under trametinib treatment, but the vulva signal is lost, and the worm is Vul on Nomarski image. Scale bars, 200 μm. **C,** Liquid cultures of the same strain imaged using a plate reader. In the magnified panels, arrows indicate adult-sized pharynges, the asterisk indicates a larval pharynx, and the arrowhead indicates a vulva. Scale bars, 500 μm.

MAPK/ERK signaling promotes vulva cell induction in *C. elegans*, and pathway inhibition can cause a Vul phenotype. We exposed WT animals to 10 μmol/L of the MEK inhibitor trametinib from the first larval stage until adulthood. Strikingly, trametinib precluded all vulva development and seemed to restrict larval growth in a fraction of the animals (Fig. 2A).

To easily discriminate between WT and Vul phenotypes, we made use of fluorescently labeled tissues. The ST65 (ncIs13) strain expresses an integrated translational GFP reporter of apical junction molecule 1 (*ajm-1*) and displays strong GFP expression in vulval and pharyngeal tissues (29). These two tissues differ in MAPK/ERK signaling dependence during development, in which vulval cell specification requires MAPK/ERK signaling and pharyngeal muscle cell specification does not. We developed a reproducible liquid culture protocol (methods) in a 96-well plate format, in which we dispensed an average of 25.5 (SD = 7.4; range, 5–66) animals per well. We grew ST65 animals in DMSO control or 10 μmol/L trametinib for 5 days, when they reached adulthood. At the first day of adulthood, we imaged plates and scored vulval and pharyngeal GFP signals manually. Vulval development was readily scored both by compound microscopy (Fig. 2B) and using photos from a fluorescence plate reader (Fig. 2C), in which the lack of mid-body GFP foci in adults corresponded to Vul phenotypes. The MAPK-independent development of pharyngeal muscle cells was unaffected, and GFP expression was retained in these cells.

**Figure 3:**
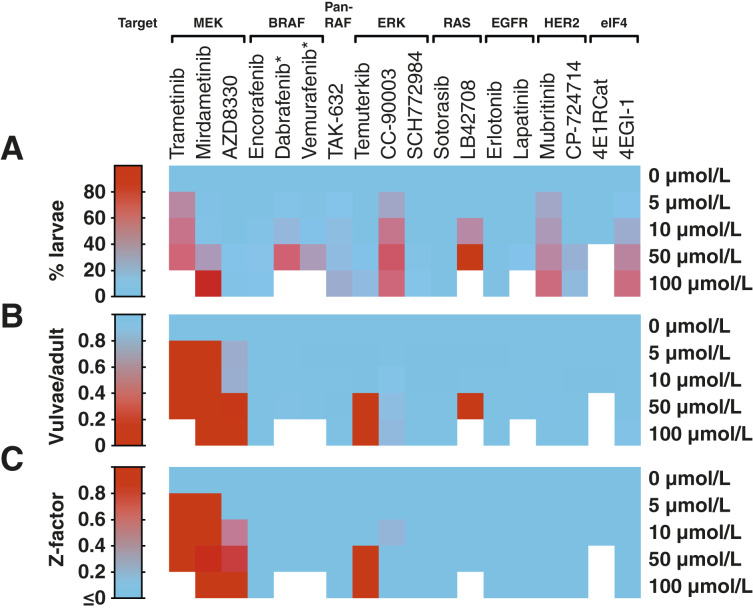
Readouts for toxicity and MAPK/ERK pathway inhibition from fluorescence-based scoring. Heatmaps showing means from three independent experiments for each drug and concentration. **A,** Putative toxicity measured as percent larvae at time of analysis. **B,** MAPK/ERK pathway inhibition as mean number of vulvae per adult worm. **C,** Assay screening performance, estimated by Z-factors. *Dabrafenib and vemurafenib were assayed up to 20 μmol/L instead of 50 μmol/L, which was nonpermissive for larval growth.

We next tested the performance of fluorescence-based scoring in a screening-like setting against a panel of 18 MAPK/ERK pathway inhibitors (Fig. 3) in concentrations up to 100 μmol/L. We imaged the plates on a Trophos Plate Runner HD machine and manually annotated the number of larvae, adults, and vulval GFP foci in each image.

In *C. elegans*, the larval growth rate has previously been used to predict compound toxicity in mammals (30, 31). We detected dose-dependent effects on growth, and within each drug class, the growth profiles varied among compounds (Fig. 3A). Among the MEK inhibitors, trametinib strongly suppressed larval growth at low concentrations, mirdametinib at intermediate concentrations, and AZD8330 hardly showed any growth suppression. Some drugs completely precluded growth at high concentrations: BRAF inhibitors dabrafenib and vemurafenib >20 μmol/L and the farnesyl transferase inhibitor LB42708 >50 μmol/L. For trametinib, we were only able to run experiments up to 50 μmol/L due to low stock concentration. Lapatinib and 4E1RCat formed autofluorescent precipitates that precluded analysis >50 and >10 μmol/L, respectively.

We found that the MEK inhibitor trametinib completely abolished vulva formation already at 5 μmol/L (Fig. 3B; Supplementary Fig. S1). Similarly, only 1.7% of animals treated with 5 μmol/L mirdametinib formed a vulva. The third MEK inhibitor, AZD8330, had a weak effect at low concentrations, but only 3.8% formed a vulva at 50 μmol/L (Supplementary Fig. S1). The ERK inhibitor temuterkib strongly inhibited vulva formation at 50 to 100 μmol/L. This was contrasted by a weak effect for the second ERK inhibitor, CC-90003. These two also differed in putative toxicity profiles, in which CC-90003 inhibited growth already at 5 μmol/L whereas temuterkib induced only minor growth effects and at high concentrations (Fig. 3A).

**Figure 4:**
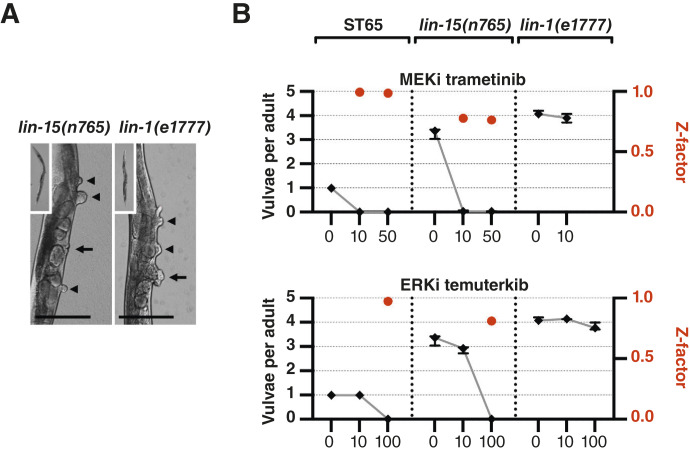
Evaluation of pathway specificity. **A,** Differential interference contrast (DIC) micrographs showing overall body morphology (inlayed panels) and vulval phenotypes in untreated *lin-15(n765)* and *lin-1(e1777)* mutant adults. Arrows indicate functional vulvae, and arrowheads indicate pseudovulvae. Scale bars, 50 μm. **B,** Graphs indicating vulvae per adult scores (black squares) and Z-factors (red dots) after drug treatment in ST65, *lin-15(n765)*, and *lin-1(e1777)* animals. Drug concentrations in μmol/L are indicated on the *X*-axis, the mean number of vulvae per adult on the left *Y*-axis, and Z-factors on the right *Y*-axis. Error bars show ± SDs from three independent experiments.

To evaluate the detection potential of the model in a high-throughput setting, we calculated effect sizes (Z-factors) for the tested compounds. Z-factors between 0.5 and 1.0 are generally considered relevant for screening, in which 1.0 indicates a perfect assay (32). Manual evaluation of adult worms and vulval GFP signals gave near-perfect Z-factors already at 5 μmol/L for the MEK inhibitors trametinib and mirdametinib with *Z* = 0.99 and 0.97, respectively (Figs. 3C and 4B). AZD8330 required higher concentrations to obtain relevance, with *Z* = 0.51 at 10 μmol/L and *Z* = 0.97 at 100 μmol/L. The assay produced Z factors of 0.98 and 0.97 for the ERK inhibitor temuterkib at 50 and 100 μmol/L, respectively. The second ERK inhibitor, CC-90003, produced a marginal assay at 10 μmol/L, whereas all other drugs produced negative Z-factors.

### MAPK/ERK pathway specificity

Ideally, drug screens for pathway-specific inhibitors would include ways to characterize on- and off-target effects at an early stage. To test if this could be achieved in *C. elegans*, we took advantage of mutations in signaling-suppressing genes upstream and downstream of the anticipated target. The *lin-15(n765)* mutation results in pathway activation upstream of the receptor LET-23/EGFR (33) and was thus expected to respond to treatment of targets downstream of the receptor. In these mutants, 99.8% (SD = 0.6, *N* = 21 experiments) of the animals displayed the Muv phenotype when grown in liquid culture (Fig. 4A). On average, *lin-15(n765)* mutants had 3.27 vulvae per adult. The Muv phenotype of *lin-15(n765)* was suppressed by both the MEK inhibitor trametinib and the ERK inhibitor temuterkib (Fig. 4B).

LIN-1 (Elk-1) is an Erythroblast Transformation Specific (ETS) domain–containing transcription factor that suppresses signaling downstream of MPK-1/ERK (34). When grown in liquid cultures, 99.8% (SD = 0.56, *N* = 21 experiments) of *lin-1(e1777)* loss-of-function mutants became Muv (Fig. 4A), with an average of 4.11 vulvae per adult worm. Drugs targeting proteins upstream of LIN-1/Elk-1 were not expected to affect vulval induction in these mutants. Accordingly, neither trametinib nor temuterkib suppressed the Muv phenotype in *lin-1(e1777)* mutants (Fig. 4B). This indicates that the phenotype suppression from drug treatment in other genetic backgrounds was likely due to pathway inhibition upstream of LIN-1. Thus, counter-screening using *lin-15(n765)* and *lin-1(e1777)* mutants demonstrated effects at the expected genetic level, and *lin-1(e1777)* mutants can be used to indicate pathway specificity.

### Automated scoring of toxicity and vulval induction

**Figure 5:**
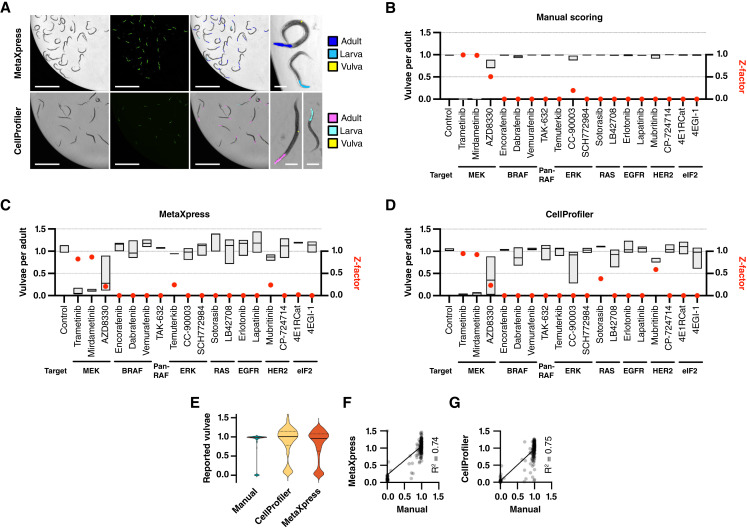
*In silico* scoring pipelines. **A,***In silico* scoring of fluorescent foci using MetaXpress and CellProfiler software. From left, brightfield images, GFP images, and the concomitant detection mask that shows each detected feature in different colors (scale bars, 1 mm). Enlarged panels to the right show examples of what each program scores as adult pharynges, larval pharynges, and vulvae (scale bars, 100 μm). **B–D,** Boxplots showing scoring of the number of vulvae per adult worm after 10 μmol/L drug exposure. Boxes represent minimum to maximum distributions of three independent experiments with a line at the median from (**B**) manual scoring, (**C**) MetaXpress, and (**D**) CellProfiler pipelines. **E,** Violin plot comparing the number of reported vulvae per adult at 10 μmol/L drug exposure from MetaXpress, CellProfiler, and manual scoring protocols. **F** and **G,** Regression analysis of the number of reported vulvae per adult at 10 μmol/L drug exposure using (**F**) MetaXpress vs. manual scoring pipelines and (**G**) CellProfiler vs. manual scoring pipelines.

Although the manual scoring protocol performed extremely well for MEK inhibitors and one ERK inhibitor, the scoring was tedious and unsuited for high-throughput settings. To meet this drawback, we compared manual scoring (Fig. 5B) with two different automated scoring protocols: one relying on proprietary software (MetaXpress) and the other on open-source software (CellProfiler). In both cases, we imaged ST65 animals on a high-content plate imaging system and trained each software to quantify (i) the number of vulva-sized foci per well, (ii) the number of adult-sized pharynges per well, and (iii) the number of non–adult-sized pharynges per well (Fig. 5A). These parameters allowed us to quantify the average number of vulvae per adult worm, the fraction of larvae, and the assay Z-factor in each condition. Each 96-well plate was imaged in around 7 minutes, resulting a theoretical throughput of 800 compounds per hour. Both automated scoring protocols detected inhibitors that significantly reduced vulval induction (Fig. 5C and D).

With the MetaXpress scoring protocol, exposure to 10 μmol/L of the MEK inhibitors trametinib and mirdametinib generated Z-factors of 0.83 and 0.87, respectively, indicating excellent assays in this automated setting. At this concentration, the third MEK inhibitor, AZD8330, the ERK inhibitor temuterkib, and, by contrast to the manual scoring protocol, the HER2 inhibitor mubritinib gave positive Z-factors. Full dose–response analyses showed that Z-factors peaked at 10 μmol/L for trametinib, mirdametinib, and mubritinib and at 100 μmol/L for AZD8330 and temuterkib (Supplementary Fig. S2A).

With manual scoring, we never observed an average of more than one vulval structure per adult. The MetaXpress scoring protocol generally overestimated the number of vulvae per adult, both after drug treatment and, notably, in DMSO controls (Fig. 5C; Supplementary Fig. S3). The CellProfiler protocol improved vulval scoring somewhat and resulted in less spread between experimental repeats in the DMSO control (Fig. 5D and E). Consequently, Z-factors improved for MEK inhibitors at 10 μmol/L (0.95 for trametinib and 0.93 for mirdametinib). The RAS inhibitor sotorasib now gave a false positive Z-factor for a higher vulval induction rate than the negative control. Full dose-response analyses revealed somewhat higher Z-factors for CellProfiler over MetaXpress but with similar peak distributions between concentrations (Supplementary Fig. S2B). The MEK inhibitor AZD8330 and the ERK inhibitor temuterkib gave maximum Z-factors of 0.87 and 0.86 at 100 μmol/L, respectively.

Under regression analysis, both *in silico* scoring pipelines gave similar R^2^ values at 10 μmol/L compared with manual vulva scoring (Fig. 5F and G). By contrast, assuming that the manual pipeline was the golden standard for vulva scoring, the CellProfiler pipeline (*R*^2^ = 0.732) outperformed the MetaXpress pipeline (*R*^2^ = 0.577) when analyzing all datapoints from all conditions tested (Supplementary Fig. S3B and S3D). Both pipelines were sensitive to fluorescent speckles from progeny, bacteria, and some drugs at high concentrations.

Both MetaXpress and CellProfiler pipelines deviated from manual scoring of larval growth (Fig. 5A; Supplementary Fig. S4A and S4C). MetaXpress overestimated the larval fraction with a baseline of 26% in DMSO controls (Supplementary Fig. S4B). CellProfiler, on the other hand, gave a lower baseline but tended to underestimate larval fractions (Supplementary Fig. S4D). By comparison, MetaXpress larval scores were more widely distributed than those of CellProfiler (Supplementary Fig. S4E) and outperformed CellProfiler with *R*^2^ values of 0.548 and 0.394, respectively (Supplementary Fig. S4B and S4D).

### A blinded screen to identify MAPK/ERK pathway inhibitors

**Figure 6:**
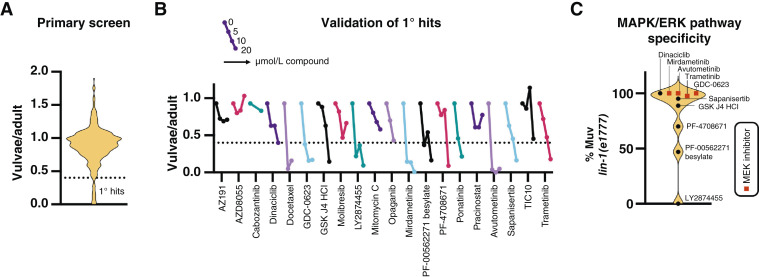
Blinded screen identification of MAPK/ERK pathway inhibitors. **A,** Violin plot showing the vulvae/adult score distribution of screened compounds after applying exclusion criteria. **B,** Hit validation of 1° hits. The plot shows the mean values for duplicate experiments. **C,** Violin plot showing percent adults with the Muv phenotype after treatment with validated 1° hits in *lin-1(e1777)* mutants. Compound names after unmasking are indicated in **B** and **C**.

Thus far, we had developed fluorescence-based scoring protocols for both potential toxicity and vulval induction as well as a counter screen to determine on–MAPK/ERK pathway and off–MAPK/ERK pathway effects with the *lin-1(e1777)* mutant. Based on the excellent Z-factors for MEK inhibitors, we proceeded with a validation screen in ST65 animals. The aim was to test if the model could identify known MEK inhibitors in a blinded screen of 433 annotated oncology-related compounds. These were tested at 10 μmol/L with DMSO as a negative control and 7 μmol/L trametinib as a positive control (see Supplementary Table S1 for the full unblinded list of tested compounds). Using the MetaXpress pipeline, we first removed compounds that gave highly overestimated vulval scores (sign of fluorescence from progeny, bacteria, or drugs) and high larval counts (potentially toxic). Out of the remaining compounds (*N* = 367), 20 compounds with vulvae/adult scores <0.4 were considered primary hits (Fig. 6A). Primary hits were then validated at 5, 10, and 20 μmol/L in ST65 animals in two independent experiments (Fig. 6B). We then reapplied the selection criteria used in the primary screen to remove wells with progeny, high larval counts, and low effect on vulval induction (>0.4 vulvae/adult in all concentrations). To determine MAPK/ERK pathway specificity, we tested the remaining 12 compounds at 10 μmol/L in the *lin-1(e1777)* mutant. Two compounds resulted in retarded larval growth which precluded analysis. Of the remaining 10, three seemed to affect vulval induction in *lin-1(e1777)* mutants, indicating they acted downstream or in parallel to the ELK1 transcription factor (Fig. 6C). The other seven were considered positive hits as they severely affected vulval induction in ST65 but not in *lin-1(e1777)* mutants. These were thus likely acting upstream of LIN-1/ELK1. After unmasking, we found that the entire 433 compound library contained seven MEK inhibitors in total. Among these, three were not identified in the screen. These were binimetinib, selumetinib, and cobimetinib. To check why these three compounds failed detection, we analyzed captured photos from the high-content plate imaging system manually. Indeed, all three produced adult worms with developed vulvae. Four of the MEK inhibitors were among the final hits. These were mirdametinib, GDC-0623, trametinib, and avutometinib, a dual RAF/MEK inhibitor. In addition, the screen identified the cyclin-dependent kinase inhibitor dinaciclib, the H3K27 histone demethylase prodrug GSK J4 HCl, and the mTor inhibitor sapanisertib as positive hits. In summary, our results demonstrate that *in vivo* drug screens based on *C. elegans* vulval development can detect clinically relevant inhibitors of MAPK/ERK signaling.

## Discussion

We have developed an assay pipeline for high-content MAPK/ERK inhibitor screens based on the vulva development model in *C. elegans.* The assay produced large effect sizes and excellent Z-factors for several clinically relevant inhibitors. This suggests that novel inhibitors with clinical relevance can be identified in nematode drug screens, in which actionable MAPK/ERK pathway proteins are highly conserved. The throughput was scalable up to 800 compounds per hour, making the assay highly suitable for discovery screens *in vivo.*

The methods used herein can be used as a reference and guide investigators to choose appropriate drug discovery assays for specific MAPK/ERK pathway targets. In such assays, trametinib would serve as an excellent positive control. Some compounds, like the ERK inhibitor temuterkib, required high concentrations to obtain relevant effect sizes. Screens for similar compounds should thus be performed at high concentrations, which in turn will cause increased rates of growth inhibition. This is not necessarily a drawback as larval growth inhibition can be used as a nonlethal endpoint in *C. elegans–*based toxicity assays (35, 36). Compounds with specific effects at high concentrations without concomitant effects on growth may thus be more relevant.

Three of the seven MEK inhibitors included in the screened chemical library failed detection. We note that IC_50_ values for human MEK1 and MEK2 were generally higher for these than for the four that were detected (37). Additionally, technical variables such as storage degradation and plating errors may have contributed to loss of effects.

Besides MEK inhibitors, the blinded validation screen identified dinaciclib, sapanisertib, and GSK-J4-HC1 targeting cyclin-dependent kinase, mTor, and H3K27 histone demethylase, respectively. In previous studies, dinaciclib reduced phosphorylation of MAPK/ERK in chronic lymphocytic leukemia cells (38), and GSK-J4-HC1 had the same effect in a zebrafish model (39). Sapanisertib has been evaluated in combination with MEK inhibitors (40); however, to our knowledge, it has not been shown to suppress MAPK/ERK signaling.

Previous studies have demonstrated that WT *C. elegans* receptor tyrosine kinases, such as LET-23/EGFR, are inherently refractory to tyrosine kinase inhibitors (25–27). Sotorasib inhibits activated KRAS^G12C^ with no inhibitory effect on WT RAS (41), and LB42708 is a farnesyl transferase inhibitor with secondary effects on RAS. Consistently, the vulval phenotype was unaffected by EGFR and RAS inhibitors in all genetic backgrounds tested. Allele-specific RAF inhibitors such as vemurafenib, encorafenib, and dabrafenib did not affect vulval induction in ST65 animals. This was expected because ST65 is WT for *lin-45*/RAF. More surprisingly, the pan-RAF inhibitor TAK-632 had no effect despite targeting WT RAF. It might be possible that structural differences between *C. elegans* LIN-45/RAF and human RAF proteins preclude meaningful assays for human RAF inhibitors. In a previous study, worm/human LET-23/EGFR chimeras were expressed to circumvent some of these issues (25). It is also possible to express and study mutant versions of *C. elegans* MAPK/ERK pathway proteins that correspond to human oncogenes, such as *lin-45*^*V627E*^/BRAF^V600E^ (20). Our WT background nematode assay seems capable of detecting MEK inhibitors, hinting at a pharmacological conformity between worm and human MEK proteins. Biologically, it has been demonstrated that mammalian RAF can phosphorylate *C. elegans* MEK-2 and that MEK-2 in turn can phosphorylate human ERK-1 (42).

In *C. elegans*, MAPK/ERK signaling defects may result in diverse phenotypes, such as larval lethality, sex myoblast migration defects, male tail development defects, and vulval development defects (43). In this study, we used the average number vulvae per adult worm as the main readout for two reasons: First, vulval defects arising from both excessive and blocked MAPK/ERK signaling (44) are readily scored. Second, scoring of the number of adults, vulvae, and larvae also gives readouts for severe effects on growth rates, indicative of high organismal toxicity (45).

The validation screen presented here was done using proprietary hardware (ImageXpress) and software (MetaXpress) but could easily be adapted to fit other plate readers. CellProfiler software is open-source and performed excellently in our study. We suggest setting up pilot studies using our CellProfiler protocol as a starting point (see Supplementary Methods) and adapting parameters to suit the imaging conditions given by the plate reader of choice.

In conclusion, we find that the genetically conserved MAPK/ERK signaling pathway in *C. elegans* responds to different classes of targeted drugs developed against human proteins. The presented screening model gives excellent Z-factors and simultaneous readouts for potential toxicity. In our validation screen, we identified seven hits, including four known MEK inhibitors. One of these was trametinib, the same compound we used as a positive control. Novel clinically relevant MAPK/ERK inhibitors can likely be identified in future high-content screens based on our *in vivo* pipeline.

## Supplementary Material

Table S1 Supplementary table 1. All Compounds and Exclusions

Supp methods and figure legends Supplementary methods and figure legends

Figure S1 Manual scoring of vulval induction in the ST65 strain.

Figure S2 Z-factors for automated scoring protocols in the ST65 strain.

Figure S3 Automated scoring of vulval induction and percent larvae in the ST65 strain.

Figure S4 Automated scoring of larvae in the ST65 strain.
