# Supplementary material for: A Drug Discovery Pipeline for MAPK/ERK Pathway Inhibitors in Caenorhabditis elegans
Source: Cancer Res Commun. 2024 Sep 18;4(9):2454–62. doi: 10.1158/2767-9764.CRC-24-0221 (PMC11409438; doi:10.1158/2767-9764.CRC-24-0221)
Supplement: Supp methods and figure legends — Supplementary methods and figure legends [file crc-24-0221_supp_methods_and_figure_legends_suppsmsfl.pdf]

SUPPLEMENTARY METHODS

Statistics

For each genotype and drug treatment, mean values and standard deviations were calculated from at least three independent experiments. Statistical significance vs. control was tested using paired, two-tailed Student's *t*-tests.

MetaXpress analysis

Images acquired with ImageXpress were analyzed with MetaXpress, the MD software. A custom module analysis protocol was created for analyzing the GFP images only. After background removal (Top Hat; Source - GFP; Size - 3000 μm; Filter Shape - Area), the "Find Blobs" function (Approx. width, 5 μm - 3000 μm; Intensity above local background, >1500) was applied to threshold all the GFP-expressing areas of worms. This size and intensity cutoff excluded most of the unspecific green, fluorescent objects from further analyses. To allow determination of the number of vulvae, a filter mask (Filter Mask vulvae) was applied on the resulting thresholded image (Find Blobs Mask) as follows; total area: 15-500, average intensity: minimum 1200 and ellips form factor: max 2.

To distinguish adults from larvae we used a size cutoff on the pharyngeal GFP expression from the AJM-1::GFP transgene. The GFP area of larval pharynges was much smaller than those in adults. A filter mask (Filter Mask adult) with a total area range of 1500-5000 μm applied on the Find Blobs Mask resulted in vulvae/adult ratio of approximately 1 in the DMSO control wells. In some experiments the minimum value had to be adjusted slightly due to experimental variation. Visual inspection of overlays with TL60 images confirmed that GFP areas excluded from the original blobs mask by the new filter masks (vulvae and adult) were almost exclusively pharynges of larvae. Finally, total area, total count and average intensity in each filter mask was measured in the Top Hat GFP image. Analyses were run automatically using the power core server of MetaXpress and logged data was exported to

excel for further analysis. The analysis protocol file (Celextra analysis protocol.xml) is included in the supplementary materials.

CellProfiler analysis

The pipeline made use of both bright field (BF) and GFP images. The most crucial step was inversion of the BF signal at the beginning of analysis. Worms were then detected based on inverted BF-photos and masks were created for detected worms. Mask were created with use of the Otsu thresholding method (settings: global, three classes, foreground) and the diameter of detected objects was set to 50 - 1000 pixels in diameter. Obtained masks were then used on GFP-images, where GFP-positive areas outside of masks were removed. Like the MetaXpress pipeline, three groups of GFP-signals were detected, although here the pipeline instead used the diameter of objects as cutoff criteria: Adult pharynges were set to 28 - 70 pixels, larval pharynges to 16 – 26 pixels, and vulvae to range 5 - 15 pixels. Obtained data was exported as .csv files and was further processed in Microsoft excel software. The analysis protocol file (worms2.cpproj) is included in the supplementary materials.

SUPPLEMENTARY FIGURE LEGENDS

Figure S1. Manual scoring of vulval induction in the ST65 strain.

Bars indicate mean numbers of vulvae per adult worm from three independent experiments. Error bars show +/- standard deviation of the mean. Statistical significance was calculated using paired, two-tailed students t-tests vs DMSO control (0 μM).

Figure S2. Z-factors for automated scoring protocols in the ST65 strain.

Heatmaps indicating vulval development inhibition Z-factors for each drug and concentration using A) MetaXpress and B) CellProfiler scoring pipelines.

Figure S3. Automated scoring of vulval induction and percent larvae in the ST65 strain. A) Heatmap generated from mean numbers of vulvae per worm from three independent experiments using the MetaXpress scoring pipeline. B) Mean number of vulvae per adult in each experimental repeat, measured and plotted on the X-axis for manual scoring and on the Y-axis for MetaXpress scoring. CD) Heatmap and plot generated as in AB) but from CellProfiler measurements instead of MetaXpress measurements. E) Violin plot generated from all vulva datapoints from MetaXpress, CellProfiler and manual scoring pipelines.

Figure S4. Automated scoring of larvae in the ST65 strain. A) Heatmap showing mean percent of larvae from three independent experiments, measured using the MetaXpress scoring pipeline. B) Mean percent larvae in each experimental repeat, plotted on the X-axis for manual scoring and on the Y-axis for MetaXpress scoring. CD) Heatmap and plot generated as in AB) but using CellProfiler measurements instead of MetaXpress measurements. E) Violin plot generated from all larval percentage datapoints, measured with MetaXpress, CellProfiler and manual scoring pipelines respectively.
