# Supplementary figures and images for: A Drug Discovery Pipeline for MAPK/ERK Pathway Inhibitors in Caenorhabditis elegans
Source: Cancer Res Commun. 2024 Sep 18;4(9):2454–62. doi: 10.1158/2767-9764.CRC-24-0221 (PMC11409438; doi:10.1158/2767-9764.CRC-24-0221)

Figure S1

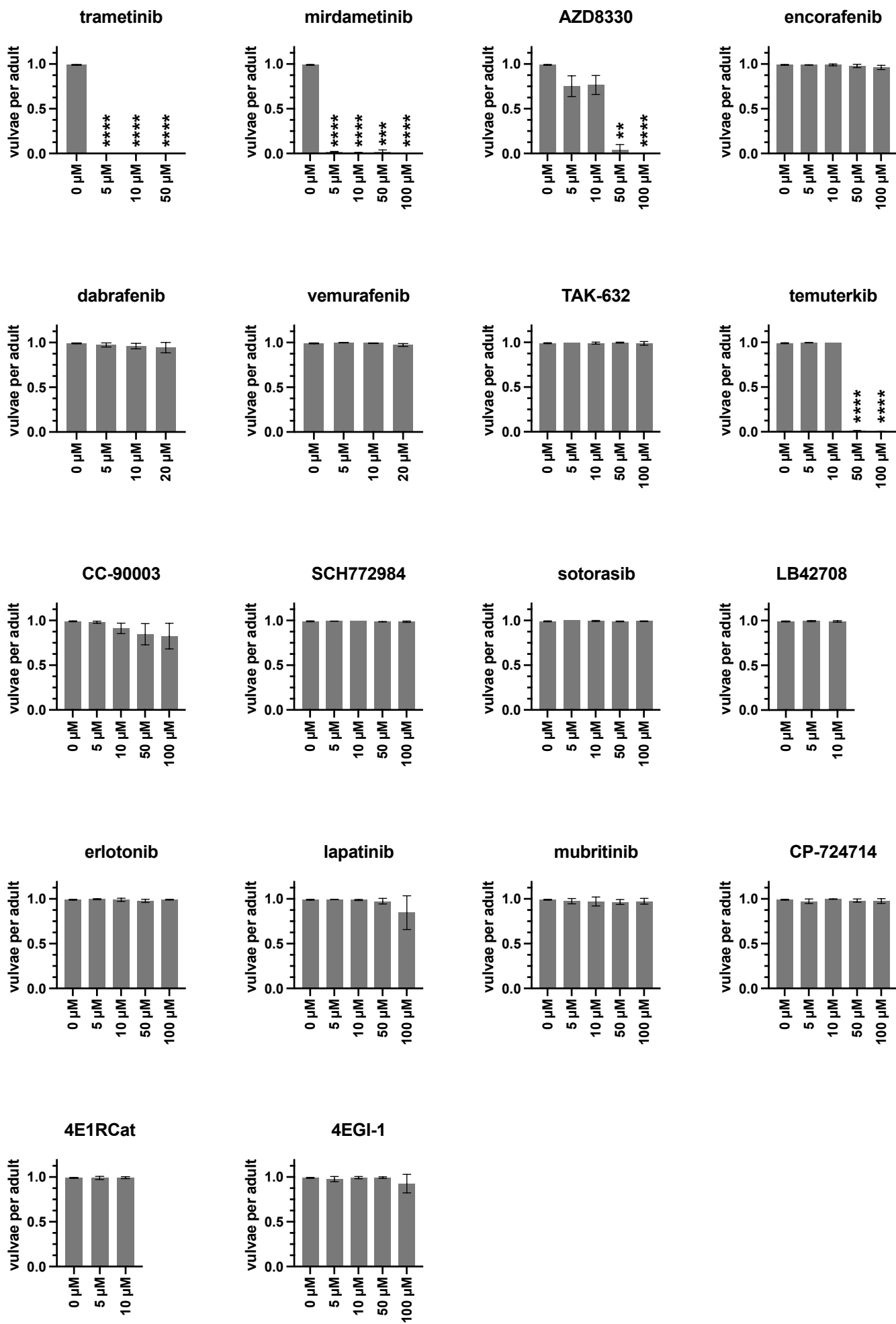

Supplement: Figure S1 — Manual scoring of vulval induction in the ST65 strain. [file crc-24-0221_figure_s1_suppsf1.pdf]

Figure S2

**A**

**MetaXpress**

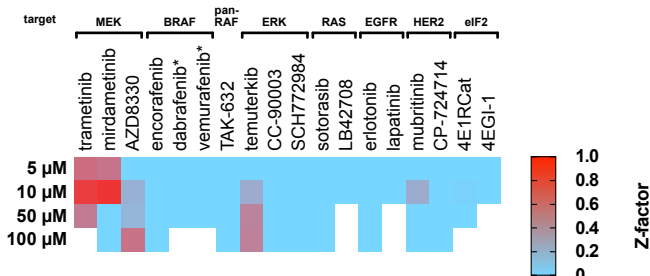

**B**

**CellProfiler**

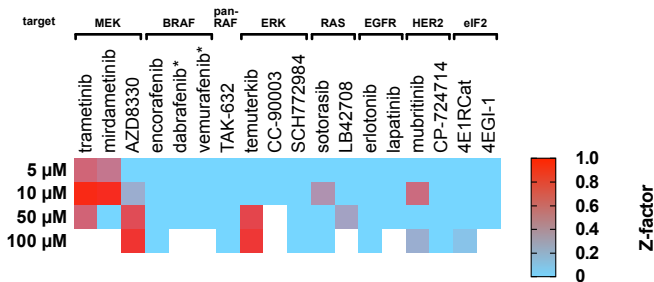

Supplement: Figure S2 — Z-factors for automated scoring protocols in the ST65 strain. [file crc-24-0221_figure_s2_suppsf2.pdf]

Figure S3

A

### MetaXpress

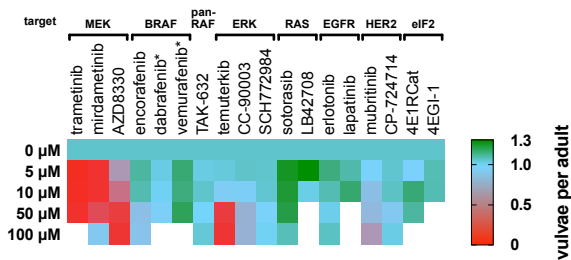

B

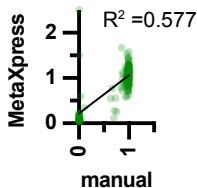

C

### CellProfiler

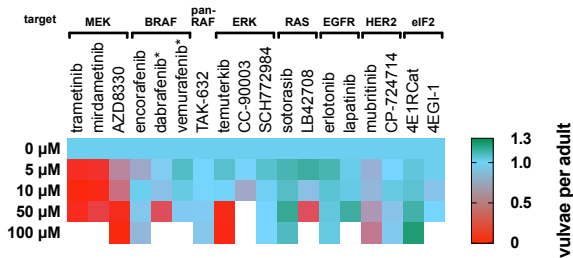

D

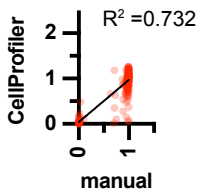

E

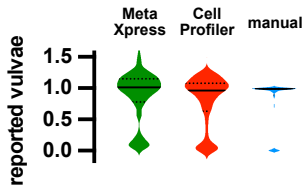

Supplement: Figure S3 — Automated scoring of vulval induction and percent larvae in the ST65 strain. [file crc-24-0221_figure_s3_suppsf3.pdf]

Figure S4

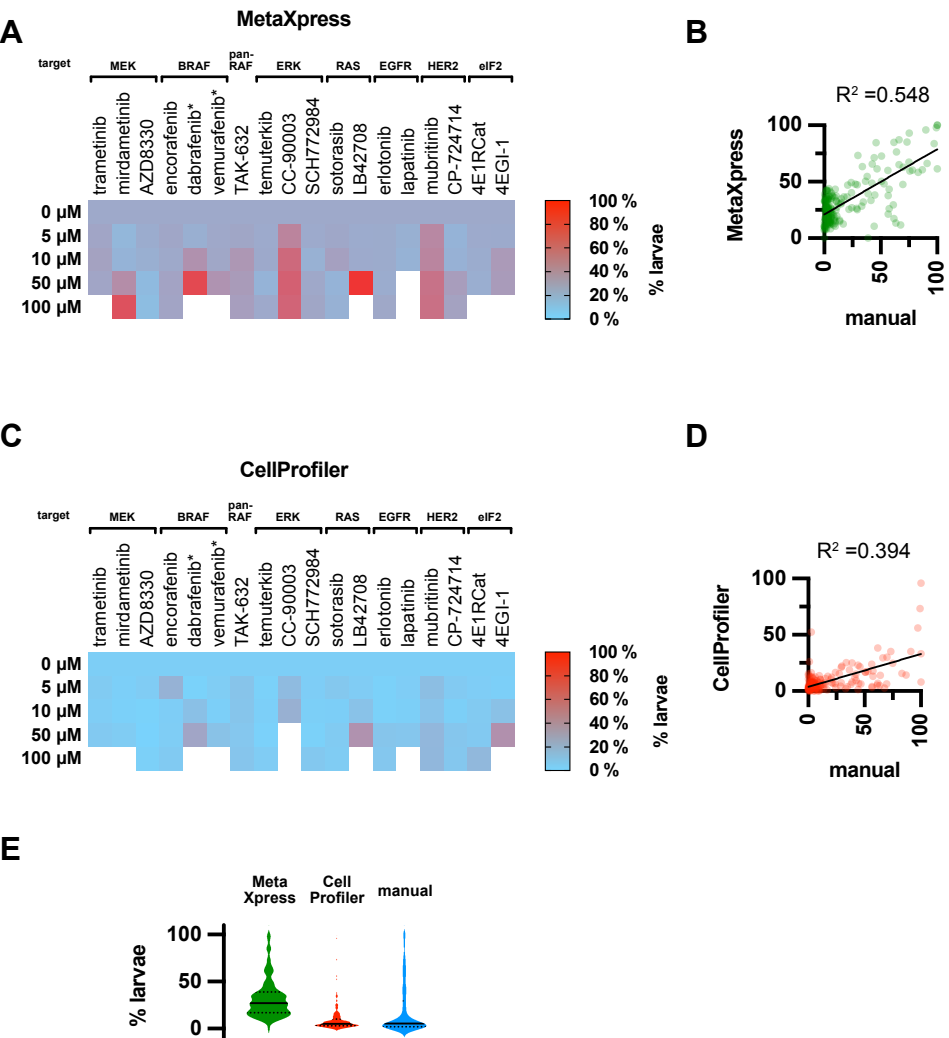

Supplement: Figure S4 — Automated scoring of larvae in the ST65 strain. [file crc-24-0221_figure_s4_suppsf4.pdf]
